# Estimation of renal function by three CKD-EPI equations in Chinese HIV/AIDS patients: A STROBE-compliant article

**DOI:** 10.1097/MD.0000000000026003

**Published:** 2021-06-04

**Authors:** Naxin Zhao, Zhili Zeng, Hongyuan Liang, Fang Wang, Di Yang, Jiang Xiao, Meiling Chen, Hongxin Zhao, Fujie Zhang, Guiju Gao

**Affiliations:** a Department of Nephrology; b Clinical and Research Center of Infectious Diseases; c Department of Medical Records and Statistics, Beijing Ditan Hospital, Capital Medical University, China.

**Keywords:** cystatin C, glomerular filtration rate, human immunodeficiency virus/acquired immune deficiency syndrome, serum creatinine

## Abstract

Assessing renal function accurately is important for human immunodeficiency virus (HIV)/acquired immune deficiency syndrome (AIDS) patients. Chronic Kidney Disease Epidemiology Collaboration (CKD-EPI) recommended three equations to calculate estimated glomerular filtration rate (eGFR). There is evidence that eGFR based on the combination of serum creatinine and cystatin C is the most accurate of the three equations. But there is limited data on the comparison of three CKD-EPI equations in Chinese HIV/AIDS patients. The aim of our study was to compare the three CKD-EPI equations in Chinese HIV/AIDS population and assess renal function.

Cross-sectional, single center, prospective study.

One hundred seventy two Chinese adult HIV/AIDS patients were enrolled, including 145 (84.3%) males and 27 (15.7%) females. Mean age was 40(±12) years old. Overall mean eGFR based on serum creatinine, cystatin C and the combination of the 2 markers was 112.6(±19.0) mL/min/1.73 m^2^, 92.0(±24.2)mL/min/1.73 m^2^, and 101.7(±21.8)mL/min/1.73 m^2^, respectively (*P* = .000). The eGFR calculated by serum creatinine alone is higher than eGFR calculated by combination of serum creatinine and cystatin C, and eGFR calculated by cystatin C individual is lower than eGFR calculated by combination of the 2 markers.

Of the 3 CKD-EPI equations, the CKD-EPI_scr-cys_ equation may have the most accuracy in evaluating renal function in Chinese HIV/AIDS patients while the CKD-EPI_scr_ equation may overestimate renal function and the CKD-EPI_cys_ equation may underestimate renal function.

## 1 Introduction

Renal dysfunction is common in human immunodeficiency virus (HIV)/acquired immune deficiency syndrome (AIDS) patients and a risk factor for poor prognosis of these patients.^[1–4]^ Assessing renal function accurately in the HIV/AIDS patients is essential, because we need to adjust the drug dosage according to kidney function. Gold standard of measuring glomerular filtration rate (GFR) is testing the clearance of inulin, iohexol, or ^99^ Tc ^m^ -diathylenetriamine pentaacetic acid, but it^,^s so cumbersome that we rarely use it in clinical settings.^[5]^ Chronic Kidney Disease Epidemiology Collaboration (CKD-EPI) recommended 3 equations to calculate estimated glomerular filtration rate (eGFR) for clinical applications in 2012^[6]^: eGFR_scr_, eGFR_cys,_ and eGFR_scr-cys_ based on serum creatinine, cystatin C and the combination of the 2 markers respectively.

Prior studies indicated that, the sensitivity of eGFR equations based on serum creatinine is poor in HIV/AIDS patients, because serum creatinine values in these patients are significantly lower than general population.^[7,8]^ In addition, a clinical study in USA demonstrated HIV-RNA > 400 copies/mL or lower CD4+ T cell count can lead to larger bias of eGFR based on cystatin C.^[9]^ A meta-analysis revealed that serum cystatin C is a better biomarker for the diagnosis of CKD in the West than in Asia.^[10]^ Inker LA found eGFR based on the combination of serum creatinine and cystatin C was more accurate than eGFR based on serum creatinine or cystatin C individually in HIV-positive population.^[11]^

Until now, there is limited data on the comparison of 3 CKD-EPI equations in Chinese HIV/AIDS patients. The aim of our study was to contrast the 3 CKD-EPI equations in Chinese HIV/AIDS population in a single center and evaluating renal function in these patients.

## 2 Methods

### 2.1 Study population

This was a cross-sectional prospective study on HIV/AIDS patients. A total of 190 Chinese HIV/AIDS patients admitted to Beijing Ditan Hospital from February to May 2019 were observed. Exclusion criteria were

1. younger than 18 years old;
2. the patients who had missing data.

Clinical information was collected from electronic medical records. The Ethics Committee of Beijing Ditan Hospital, Capital Medical University approved the study protocol. The approval numbers is Jdlkz 2019-056-02. We obtained written informed consent from each subject.

### 2.2 Laboratory measurements and eGFR equations

Serum creatinine was measured at Department of Clinical Laboratory in Beijing Ditan Hospital affiliated to Capital Medical University using enzymatic assay. Collected 3.5 ml fasting venous blood of eligible subjects, took serum after centrifugation for 15 min under 3000 r /min, conserved the serum in −80°C. Finally, remelted the frozen serum together for testing cystatin C. The cystatin C test reagent kit was produced by Zhangjiagang DIALAB Biotechnology Co. Ltd, China. Serum cystatin C was measured with automatic biochemical analyzer HITACHI 7600 using a latex enhanced immunoturbidimetric assay. The eGFR was calculated by 3 CKD-EPI equations^[6]^ (Table 1).

**Table 1:** Three CKD-EPI equations for eGFR.

Subjects	Gender	Scr (mg/dL)	Scys (mg/L)	Equation (mL/min/1.73 m^2^)
eGFR_scr_	female	≤0.7		141 × (Scr/0.7)^−0.329^ × 0.993^age^ × 1.018
		>0.7		141 × (Scr/0.7)^−1.209^ × 0.993^age^ × 1.018
	male	≤0.9		141 × (Scr/0.9)^−0.411^ × 0.993^age^
		>0.9		141 × (Scr/0.9)^−1.209^ × 0.993^age^
eGFR_cys_	female		≤0.8	133 × (Scys/0.8)^−0.499^ × 0.996^age^ × 0.932
			>0.8	133 × (Scys/0.8)^−1.328^ × 0.996^age^ × 0.932
	male		≤0.8	133 × (Scys/0.8)^−0.499^ × 0.996^age^
			>0.8	133 × (Scys/0.8)^−1.328^ × 0.996^age^
eGFR_scr-cys_	female	≤0.7	≤0.8	130 × (Scr/0.7)^−0.248^ × (Scys/0.8)^−0.375^ × 0.995^age^
			>0.8	130 × (Scr/0.7)^−0.248^ × (Scys/0.8)^−0.711^ × 0.995^age^
		>0.7	≤0.8	130 × (Scr/0.7)^−0.601^ × (Scys/0.8)^−0.375^ × 0.995^age^
			>0.8	130 × (Scr/0.7)^−0.601^ × (Scys/0.8)^−0.711^ × 0.995^age^
	male	≤0.9	≤0.8	135 × (Scr/0.9)^−0.207^ × (Scys/0.8)^−0.375^ × 0.995^age^
			>0.8	135 × (Scr/0.9)^−0.207^ × (Scys/0.8)^−0.711^ × 0.995^age^
		>0.9	≤0.8	135 × (Scr/0.9)^−0.601^ × (Scys/0.8)^−0.375^ × 0.995^age^
			>0.8	135 × (Scr/0.9)^−0.601^ × (Scys/0.8)^−0.711^ × 0.995^age^

### 2.3 Statistical analysis

Data were analyzed using SPSS20.0 (SPSS Institute, Chicago IL, USA). Homogeneity of quantitative data were tested by Kolmogorov-Smirnov one-sample test. Data with normal distribution was presented as mean ±SD. If data was in abnormal distribution, it was presented as median (interquartile range). Categorical variables were prescribed as frequency and percentage. Comparisons between eGFR values calculated by 3 CKD-EPI equations (eGFR_scr_, eGFR_cys,_ and eGFR_scr-cys_) were carried out by using the Wilcoxon signed rank test. *P* values <.05 were considered statistically significant in this study.

## 3 Results

One of the 190 Chinese HIV/AIDS patients admitted to Beijing Ditan Hospital from February to May 2019 was ineligible because he was younger than 18 years old, and 17 of them were excluded because of missing values for serum cystatin C. Finally, 172 Chinese adult HIV/AIDS patients were enrolled, including 145 (84.3%) males and 27 (15.7%) females.

### 3.1 Patient characteristics

Mean age was 40(±12) years old, the youngest patient was 19 years old, and the oldest 1 was 76 years old. Mean body mass index was 22 (±4) kg/m^2^. 17 (9.9%), 13 (7.6%), 65 (37.8%), and 14 (8.1%) patients had hypertension, diabetes, dyslipidemia, and tumor, respectively. HBV, HCV and Syphilis existed in 11 (6.4%), 8 (4.7%), 43 (25.0%) patients, respectively. Median duration of HIV infection was 12 months (IQR 0–72). 44 (25.6%) of them were newly discovered cases. The longest duration of HIV-infection was 276 months. One hundred fourteen patients (66.3%) were receiving antiretroviral therapy. Sixty nine patients (40.1%) had HIV-RNA <20 copies/ml or undetectable. Median CD4 + T cell count was 120 (IQR 42–458) cells/μL. More details are described in Table 2.

**Table 2:** Main demographic characteristics of 172 HIV/AIDS patients included in the study.

Variables	Estimates
Mean age, yr (±SD)	40 (±12)
Gender, n (%)	
Male	145 (84.3%)
Female	27 (15.7%)
Mean body mass index, kg/m^2^ (±SD)	22 (±4)
Hypertension, n (%)	17 (9.9%)
Diabetes mellitus, n (%)	13 (7.6%)
Dyslipidemia, n (%)	65 (37.8%)
Tumor, n (%)	14 (8.1%)
Hepatitis B coinfection, n (%)	11 (6.4%)
Hepatitis C coinfection, n (%)	8 (4.7%)
Syphilis, n (%)	43 (25.0%)
Median duration of HIV infection, months (IQR)	12 (0–72)
Current ART regimen, n (%)	
No treatment	58 (33.7%)
TDF + 3TC + EFV	66 (38.4%)
TDF + 3TC + LPV/r	18 (10.5%)
TDF + 3TC + DTG	5 (2.9%)
EVG/C/TAF/FTC	5 (2.9%)
Others^∗^	20 (11.6%)
Current HIV infection status, n (%)	
Suppression under treatment (viral load <20 copies/ml)	69 (40.1%)
No suppression (including no treatment)	103 (59.9%)
Median CD4+ T cell count, cells/μL (IQR)	120 (42–458)

### 3.2 eGFR calculated by 3 CKD-EPI equations

The overall mean eGFR based on serum creatinine, cystatin C and the combination of the 2 markers was 112.6(±19.0) mL/min/1.73 m^2^, 92.0(±24.2) mL/min/1.73 m^2^, and 101.7(±21.8) mL/min/1.73 m^2^, respectively (*P* = .000). The differences of mean eGFR by the 3 equations were provided in Table 3.

**Table 3:** Differences of mean eGFR calculated by 3 CKD-EPI equations.

Comparation of variables	Difference of mean (mL/min/1.73 m^2^)	*P* value^∗^
eGFR_scr_ - eGFR_cys_	20.6	.000
eGFR_scr_ - eGFR_scr-cys_	10.9	.000
eGFR_cys_- eGFR_scr-cys_	−9.7	.000

The frequencies of patients in each eGFR category are described in Table 4. If we use different equations to calculate eGFR, we can see that the frequencies and percentages of patients in each eGFR category is different. Generally, we can see that, eGFR calculated by serum creatinine is higher than eGFR calculated by combination of the 2 markers, and eGFR calculated by serum cystatin C is lower than eGFR calculated by combination of the 2 markers.

**Table 4:** Frequencies and percentages of patients in each eGFR category.

eGFR category	Number (%) of patients with each eGFR category by 3 equations		
(mL/min/1.73 m^2^)	CKD-EPI_scr_	CKD-EPI_cys_	CKD-EPI_scr-cys_
≥90	154 (89.5%)	102 (59.3%)	131 (76.2%)
60–89	14 (8.1%)	53 (30.8%)	32 (18.6%)
30–59	4 (2.3%)	14 (8.1%)	8 (4.7%)
15–29	0 (0%)	3 (1.7%)	1 (0.6%)
<15	0 (0%)	0 (0%)	0 (0%)

Table 5 provided more details about the comparison of eGFR classifications between CKD-EPI_scr-cys_ equation and the other 2 equations. Of the 154 patients with eGFR_scr_≥90 mL/min/1.73 m^2^, 23 patients presented eGFR_scr-cys_ < 90 mL/min/1.73 m^2^, and 1 among them even showed eGFR_scr-cys_ < 60 mL/min/1.73 m^2^. In the 14 patients who had eGFR_scr_ located in 60-89 mL/min/1.73 m^2^, 4 of them presented eGFR_scr-cys_ located in 30-59 mL/min/1.73 m^2^. Of the 131 patients with eGFR_scr-cys_≥90 mL/min/1.73 m^2^, 30 patients had eGFR_cys_ < 90 mL/min/1.73 m^2^. Of the 53 patients with eGFR_cys_ located in 60-89 mL/min/1.73 m^2^, 30 patients had eGFR_scr-cys_≥90 mL/min/1.73 m^2^.

**Table 5:** Comparison of eGFR classifications between CKD-EPI_scr-cys_ equation and the other 2 equations.

	eGFR_scr-cys_ (mL/min/1.73 m^2^)					
N	≥90	60–89	30–59	15–29	<15	Total
eGFR_scr_ ≥90	131	22	1	0	0	154
60–89	0	10	4	0	0	14
30–59	0	0	3	1	0	4
15–29	0	0	0	0	0	0
<15	0	0	0	0	0	0
eGFR_cys_ ≥90	101	1	0	0	0	102
60–89	30	23	0	0	0	53
30–59	0	8	6	0	0	14
15–29	0	0	2	1	0	3
<15	0	0	0	0	0	0
Total	131	32	8	1	0	172

## 4 Discussion

We used enzymatic method for the determination of serum creatinine, because previous studys indicated the enzymatic method is more accurate than Jaffe method.^[12,13]^ In our study, the eGFR calculated by serum creatinine alone was 112.6(±19.0) mL/min/1.73 m^2^, which was the highest in the 3 CKD-EPI equations. And our data are consistent with prior studies.^[8,14]^ Clara et al revealed serum creatinine may overestimate renal function in HIV-infected subjects.^[8]^ In a cohort of HIV-infected women, Driver et al^[14]^ found that the prevalence of CKD was higher with eGFR_cys_ compared to eGFR_scr_. The overestimation of renal function and thus underestimating kidney impairment by serum creatinine in HIV/AIDS patients is due to decreasing serum creatinine concentrations in this population. Low muscle mass is common in HIV/AIDS patients.^[15]^ Both HIV itself and HIV antiretroviral medications could lead to muscle disease and decrease the concentration of creatinine.^[8,16–18]^

It should be noted that, although dolutegravir or rilpivirine may inhibit renal creatinine secretion, leading to an increase in serum creatinine in HIV/AIDS patients treated with these drugs,^[19,20]^ this phenomenon was not been observed in our study. Maybe it's because there are few patients taking these drugs in our study (8 patients used dolutegravir and none took rilpivirine).

Cystatin C is produced by all nucleated cells at a constant rate in the body and is less affected by muscle mass than creatinine.^[21]^ An analysis on 922 HIV-infected subjects conducted by Choi A and colleagues revealed that eGFR based on cystatin C was significantly associated with 5-year all-cause mortality, whereas eGFR based on serum creatinine did not appear to be associated with mortality substantially.^[22]^ Nevertheless, Bhasin et al found^[9]^ eGFR based on cystatin C was significantly more biased than eGFR based on combination of serum creatinine and cystatin C in the HIV-positive group, and eGFR based on cystatin C was lower than measured GFR using plasma iohexol clearance. In our present study, we found the eGFR calculated by serum cystatin C is lower than eGFR calculated by combination of serum creatinine and cystatin C [92.0(±24.2) mL/min/1.73 m^2^ vs 101.7(±21.8) mL/min/1.73 m^2^]. So our result is in accordance with Bhasin et al despite we did not measure GFR with gold standard method. That is to say, eGFR based on combination of serum creatinine and cystatin C has greater GFR fidelity while eGFR based on cystatin C is a better predictor of clinical outcomes. This is not a contradiction. eGFR based on cystatin C had strong correlations with HIV-RNA viral load, CD4+ T cell count, hs-CRP, IL-6, and D-dimer in HIV-infected persons.^[21]^ Emerging data from HIV-infected populations exhibited the strong associations between clinical events including all-cause mortality and inflammatory markers, notably IL-6 and D-dimer.^[23–25]^ Consequently, inflammation may mediate the association between eGFR based on cystatin C and clinical events.

Similar outcomes were discovered in general populations.^[26–29]^ Inker LA^[26]^ found that the eGFR equation based on combination of serum creatinine and cystatin C was significantly more accurate than the eGFR equation based on cystatin C alone. A research performed by Chi^[27]^ et al showed that the CKD-EPI _scr-cys_ equation was more suitable for estimating renal function than the other equations in a Chinese general population. Zhu Y^[29]^ also corroborated that CKD-EPI _scr-cys_ formula had better diagnostic value, especially in young participants.

Our study has following advantages. Firstly, this is the first clinical study to compare 3 CKD-EPI equations conducted in Chinese HIV/AIDS population. Secondly, we used standardized serum creatinine and cystatin C measurements.

Our study also has limitations. Firstly, we did not directly measure GFR using gold standard method. Secondly, the number of patients was relatively small. Thirdly, there was no HIV/AIDS patients with eGFR < 15 mL/min/1.73 m^2^. A prospective larger scale study comparing the performance of different eGFR formulas with gold standard of measuring GFR in Chinese HIV/AIDS patients should be conducted in the future.

## 5 Conclusion

In conclusion, of the 3 CKD-EPI equations, the CKD-EPI_scr-cys_ equation may have the most accuracy in evaluating renal function in Chinese adult HIV/AIDS patients as the CKD-EPI_scr_ equation may overestimate renal function and the CKD-EPIcys equation may underestimate renal function.

## Acknowledgments

We thank all the patients and staff in Clinical and Research Center of Infectious Diseases, Beijing Ditan Hospital. We especially appreciate Jiyun Sun and his colleague in Department of Clinical Laboratory, Beijing Ditan Hospital, for testing serum cystatin C.

## Author contributions

**Conceptualization:** Guiju Gao.

**Data curation:** Zhili Zeng.

**Formal analysis:** Meiling Chen.

**Investigation:** Hongyuan Liang, Fang Wang, Di Yang, Jiang Xiao.

**Resources:** Fujie Zhang.

**Visualization:** Guiju Gao.

**Writing – original draft:** Naxin Zhao.

**Writing – review & editing:** Hongxin Zhao, Fujie Zhang.

## References

[1] Mocroft A Kirk O Reiss P. Estimated glomerular filtration rate, chronic kidney disease and antiretroviral drug use in HIV-positive patients. AIDS 2010;24:1667–78. 20523203 10.1097/QAD.0b013e328339fe53

[2] Lucas GM Clarke W Kagaayi J. Decreased kidney function in a community-based cohort of HIV-Infected and HIV-negative individuals in Rakai, Uganda. J Acquir Immune Defic Syndr 2010;55:491–4. 20613548 10.1097/QAI.0b013e3181e8d5a8 PMC2974780

[3] Obiri-Yeboah D Awuku YA Alofa W. Renal dysfunction among adult HIV/AIDS patients on antiretroviral therapy at a tertiary facility in Ghana. BMC Nephrol 2018;19:333. 30463531 10.1186/s12882-018-1130-z PMC6249759

[4] Lopez ED Córdova-Cázarez C Valdez-Ortiz R. Epidemiological, clinical, and laboratory factors associated with chronic kidney disease in Mexican HIV-infected patients. J Bras Nefrol 2019;41:48–54. 30010693 10.1590/2175-8239-JBN-2018-0024 PMC6534026

[5] Soveri I Berg UB Björk J. Measuring GFR: a systematic review. Am J Kidney Dis 2014;64:411–24. 24840668 10.1053/j.ajkd.2014.04.010

[6] National Kidney Foundation. KDOQI clinical practice guideline for diabetes and CKD: 2012 update. Am J Kidney Dis 2012;60:850–86. 23067652 10.1053/j.ajkd.2012.07.005

[7] Seape T Gounden V van Deventer HE. Cystatin C and creatinine-based equations in the assessment of renal function in HIV-positive patients prior to commencing Highly Active Antiretroviral Therapy. Ann Clin Biochem 2016;53:58–66. 25766385 10.1177/0004563215579695

[8] Jones Clara Y Jones Camille A Wilson Ira B. Cystatin C and creatinine in An HIV cohort: the Nutrition for Healthy Living Study. Am J Kidney Dis 2008;51:914–24. 18455851 10.1053/j.ajkd.2008.01.027 PMC4430838

[9] Bhasin B Lau B Atta MG. HIV viremia and T-Cell activation differentially affect the performance of glomerular filtration rate equations based on creatinine and cystatin C. PLoS One 2013;8:e82028. 24376511 10.1371/journal.pone.0082028 PMC3871673

[10] Wei L Ye X Pei X. Diagnostic accuracy of serum cystatin C in chronic kidney disease: a meta-analysis. Clin Nephrol 2015;84:86–94. 26200185 10.5414/cn108525

[11] Inker LA Wyatt C Creamer R. Performance of creatinine and cystatin C GFR estimating equations in an HIV-positive population on antiretrovirals. J Acquir Immune Defic Syndr 2012;61:302–9. 22842844 10.1097/QAI.0b013e31826a6c4f PMC3598619

[12] Greenberg N Roberts WL Bachmann LM. Specificity characteristics of 7 commercial creatinine measurement procedures by enzymatic and Jaffe method principles. Clin Chem 2012;58:391–401. 22166253 10.1373/clinchem.2011.172288

[13] Liu WS Chung YT Yang CY. Serum creatinine determined by Jaffe, enzymatic method, and isotope dilution-liquid chromatography-mass spectrometry in patients under hemodialysis. J Clin Lab Anal 2012;26:206–14. 22628238 10.1002/jcla.21495 PMC6807401

[14] Driver TH Scherzer R Peralta CA. Comparisons of creatinine and cystatin C for detection of kidney disease and prediction of all-cause mortality in HIV-infected women. AIDS 2013;27:2291–9. 23669156 10.1097/QAD.0b013e328362e874 PMC3919542

[15] Buehring B Kirchner E Sun Z. The frequency of low muscle mass and its overlap with low bone mineral density and lipodystrophy in individuals with HIV--a pilot study using DXA total body composition analysis. J Clin Densitom 2012;15:224–32. 22169198 10.1016/j.jocd.2011.10.003

[16] Natsag J Erlandson KM Sellmeyer DE. HIV infection is associated with increased fatty infiltration of the thigh muscle with aging independent of fat distribution. PLoS One 2017;12:e0169184. 28060856 10.1371/journal.pone.0169184 PMC5218482

[17] Calza L Danese I Colangeli V. Skeletal muscle toxicity in HIV-1-infected patients treated with a raltegravir-containing antiretroviral therapy: a cohort study. AIDS Res Hum Retroviruses 2014;30:1162–9. 25369244 10.1089/aid.2014.0113

[18] Wasserman P Segal-Maurer S Rubin DS. High prevalence of low skeletal muscle mass associated with male gender in midlife and older HIV-infected persons despite CD4 cell reconstitution and viral suppression. J Int Assoc Provid AIDS Care 2014;13:145–52. 24067494 10.1177/2325957413495919

[19] Raffi F Rachlis A Stellbrink H-J. Once-daily dolutegravir versus raltegravir in antiretroviral-naive adults with HIV-1 infection: 48 week results from the randomised, double-blind, non-inferiority SPRING-2 study. Lancet 2013;381:735–43. 23306000 10.1016/S0140-6736(12)61853-4

[20] Nadia Galizzi Laura Galli Andrea Poli. Glomerular filtration rate estimated by cystatin C formulas in HIV-1 patients treated with dolutegravir,rilpivirine or cobicistat. New Microbiol 2018;41:256–61. 30252923

[21] Lucas GM Cozzi-Lepri A Wyatt CM. Glomerular filtration rate estimated using creatinine, cystatin C or both markers and the risk of clinical events in HIV-infected individuals. HIV Med 2014;15:116–23. 24024499 10.1111/hiv.12087 PMC3947332

[22] Choi A Scherzer R Bacchetti P. albuminuria, and 5-year all-cause mortality in HIV-infected persons. Am J Kidney Dis 2010;56:872–82. 20709438 10.1053/j.ajkd.2010.05.019 PMC3164880

[23] Hunt PW Lee SA Siedner MJ. Immunologic biomarkers, morbidity, and mortality in treated HIV infection. J Infect Dis 2016;214: Suppl 2: S44–50. 27625430 10.1093/infdis/jiw275 PMC5021241

[24] Borges ÁH O’Connor JL Phillips AN. Interleukin 6 is a stronger predictor of clinical events than high-sensitivity C-reactive protein or D-dimer during HIV infection. J Infect Dis 2016;214:408–16. 27132283 10.1093/infdis/jiw173 PMC4936649

[25] Grund B Baker JV Deeks SG. Relevance of interleukin-6 and D-dimer for serious non-AIDS morbidity and death among HIV-positive adults on suppressive antiretroviral therapy. PLoS One 2016;11:e0155100. 27171281 10.1371/journal.pone.0155100 PMC4865234

[26] Inker LA Schmid CH Tighiouart H. Estimating glomerular filtration rate from serum creatinine and cystatin C. N Engl J Med 2012;367:20–9. 22762315 10.1056/NEJMoa1114248 PMC4398023

[27] Chi XH Li GP Wang QS. CKD-EPI creatinine-cystatin C glomerular filtration rate estimation equation seems more suitable for Chinese patients with chronic kidney disease than other equations. BMC Nephrol 2017;18:226. 28693441 10.1186/s12882-017-0637-z PMC5504640

[28] Liu X Ma H Huang H. Is the chronic kidney disease epidemiology coll<∗∗∗>aboration creatinine-cystatin C equation useful for glomerular filtration rate estimation in the elderly? Clin Interv Aging 2013;8:1387–91. 24143084 10.2147/CIA.S52774 PMC3797613

[29] Zhu Y Ye X Zhu B. Comparisons between the 2012 new CKD-EPI (chronic kidney disease epidemiology collaboration) equations and other four approved equations. PLoS One 2014;9:e84688. 24454737 10.1371/journal.pone.0084688 PMC3890277

